# Perioperative Glucose Control in Neurosurgical Patients

**DOI:** 10.1155/2012/690362

**Published:** 2012-02-13

**Authors:** Daniel Agustín Godoy, Mario Di Napoli, Alberto Biestro, Rainer Lenhardt

**Affiliations:** ^1^Neurocritical Care Unit, Sanatorio Pasteur, Catamarca, Argentina; ^2^Neurological Service, San Camillo de' Lellis General Hospital, 02100 Rieti, Italy; ^3^Intensive Treatment Center, Hospital de Clínicas, Montevideo, Uruguay; ^4^Department of Anesthesiology and Perioperative Medicine, University of Louisville, Louisville, KY 40202, USA

## Abstract

Many neurosurgery patients may have unrecognized diabetes or may develop stress-related hyperglycemia in the perioperative period. Diabetes patients have a higher perioperative risk of complications and have longer hospital stays than individuals without diabetes. Maintenance of euglycemia using intensive insulin therapy (IIT) continues to be investigated as a therapeutic tool to decrease morbidity and mortality associated with derangements in glucose metabolism due to surgery. Suboptimal perioperative glucose control may contribute to increased morbidity, mortality, and aggravate concomitant illnesses. The challenge is to minimize the effects of metabolic derangements on surgical outcomes, reduce blood glucose excursions, and prevent hypoglycemia. Differences in cerebral versus systemic glucose metabolism, time course of cerebral response to injury, and heterogeneity of pathophysiology in the neurosurgical patient populations are important to consider in evaluating the risks and benefits of IIT. While extremes of glucose levels are to be avoided, there are little data to support an optimal blood glucose level or recommend a specific use of IIT for euglycemia maintenance in the perioperative management of neurosurgical patients. Individualized treatment should be based on the local level of blood glucose control, outpatient treatment regimen, presence of complications, nature of the surgical procedure, and type of anesthesia administered.

## 1. Introduction

Several observational and interventional studies have indicated that hyperglycemia (hyperG) in diabetic and nondiabetic neurosurgical patients is associated with adverse outcomes, such as an increased prevalence of complications, prolonged hospital stay, and higher mortality rates [1–5]. In addition there are deleterious effects of glucose deficit on brain metabolism [6, 7]. Individuals with previously unknown hyperG are at greater risk than those with preexisting diabetes mellitus (DM) [8]. Available evidence shows that hyperG has negative consequences on the whole organism, including the brain [9–12]. Undiagnosed DM and hospital-induced hyperG increase postoperative complications, hospital costs, and length of stay [13–15]. hyperG is closely linked to prognosis in different brain injury scenarios [16]. Nevertheless, no consensus exists as to whether hyperG is directly responsible for poor outcomes or if it is just an epiphenomenon of brain damage [10–12, 16]. It has been hypothesized, therefore, that strict blood glucose control could have a favorable impact on patient outcome [11]. Consequently, increasing interest has evolved for tight blood glucose control using intensive insulin therapy (IIT) in neurocritically ill patients. Tight blood glucose control has been defined as glucose controlled within a range of 80 to 110 mg/dL (4.40 to 6.10 mmol/L). However, more recent data suggest possible deleterious effects of IIT on brain tissue. To date, solid clinical evidence to justify IIT in neurocritically ill patients does not exist [4, 16]. 

Caution is necessary when generalizing IIT clinical studies from critical ill patients to neurosurgical patients since brain glucose metabolism is often altered by neurological injury. A persistent hyperG creates numerous untoward consequences, while iatrogenic hypoglycemia (hypoG) may initiate a metabolic crisis in the brain that is even worse [16]. Moreover, the upper and lower thresholds of plasma glucose for these adverse effects are not clearly defined and peripheral glucose measurements do not consistently correlate with brain glucose levels [12, 13, 16, 17].

Improving blood glucose control in the perioperative period could mitigate many of the detrimental consequences of hyperG [12, 13, 16, 17]. In diabetic patients, concomitant conditions, such as obesity, hypertension, renal insufficiency, and coronary artery disease, increase perioperative risk [13–15]. Surgery is a stressful event that leads to temporary disruption of oral intake and frequently requires adjustment of antidiabetic therapy [14, 15]. To minimize the surgical complications due to metabolic derangements and the effects of surgery on glycemic control, level of blood glucose control, outpatient treatment regimen, presence of complications, nature of the surgical procedure, inpatient glucose response, and type of anesthesia should be taken into account.

This paper focuses on perioperative glucose control in neurosurgical patients and glucose management during the perioperative period. In addition, it features a summary of guidelines for specific neurosurgical pathologies in the acute (perioperative) period.

## 2. Causes of Glucose Variations during the Perioperative Period in Neurosurgery

### 2.1. Surgical Stress and Glucose Levels

#### 2.1.1. Hyperglycemia

There is no unanimous and clear definition about hyperG or hypoG in the non-DM population, nor is it well established when to start treatment. The American Diabetes Association consensus recently established the presence of hyperG and patient treatment threshold when blood glucose values exceed 140 mg/dL (7.8 mmol/L) in two or more plasma samples [8].

**Figure 1:**
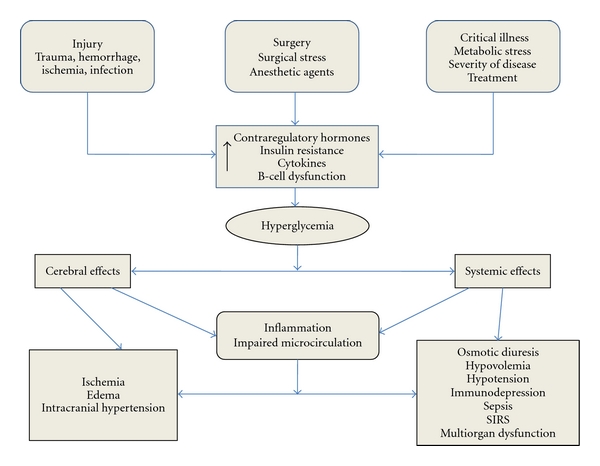
Causes and consequences of hyperglycemia. Where SIRS: Systemic inflammatory response syndrome.

The stress of surgery activates a neuroendocrine response that antagonizes the action of insulin and predisposes the patient to hyperG and ketoacidosis (Figure 1). Consequently, an increase of the secretion of counterregulatory hormones (e.g., epinephrine, cortisol) can be observed [16, 18, 19]. Stress also induces the development of insulin resistance, generated by proinflammatory cytokines [20, 21] or caused iatrogenically by commonly used drugs (e.g., dopamine, noradrenaline, corticosteroids, thiazides, and dextrose-containing solutions) [9, 18, 22]. Stress-induced hyperG may cause endothelial cell dysfunction, defects in immune function, increased oxidative stress, prothrombotic changes, cardiovascular effects, and specific brain area (insular cortex) injury or a direct hypothalamic damage/irritation of glucose regulatory centers [23–25]. hyperG has been shown to aggravate these deleterious effects, whereas optimization of glucose control has been shown to reverse them.

#### 2.1.2. Hypoglycemia

hypoG is defined as a blood glucose value of <70 mg/dL (<3.9 mmol/L) [8]. hypoG is a common, multifactorial, and avoidable event. It can occur under any circumstances, although the DM population is the most susceptible. There are no specific data about its incidence in neurosurgical patients, but it is well known that hypoG events worsen prognosis [13, 26, 27]. The hypoG-associated risk is greater during the perioperative period, when general anesthesia may mask the symptoms and delay its recognition.  Table 1 shows the most common causes of hypoG in these patients.

### 2.2. Surgical Risks and Benefits of Improved Glucose Control

Patients with diabetes have more comorbidities compared with the general population, such as obesity, hypertension, sleep apnea, cardiovascular disease, congestive heart failure, undetected atherosclerosis (coronary, cerebral, and peripheral), and renal insufficiency. Diabetic autonomic neuropathy (advanced cardiac, respiratory, and gastrointestinal autonomic neuropathy) can lead to hemodynamic instability, abnormal gut motility, and erroneous glucose levels. In addition, inadequate glucose control leads to increased risk of infectious complications [14, 15].

The benefits of improved blood glucose control after neurosurgery include a lower rate of craniotomy wound infections, reduced length of stay, and reduced hospital cost [14, 15]. Decreased bloodstream and nosocomial infections, acute renal failure, ventilatory support, blood transfusions, critical illness polyneuropathy, and duration of stay in the neurocritical intensive care unit have also been demonstrated [5, 28, 29].

### 2.3. Diabetes and Increased Risk of Adverse Outcomes in Neurosurgery

DM is a risk factor for suboptimal perioperative outcomes in patients undergoing neurosurgery [15, 30]. Several studies have identified an association between diabetes and infectious complications after major spine surgery [31]. Patients with DM also have an increased frequency of complications, both major (e.g., wound infection, peripheral nerve root lesion, cardiac arrhythmia, acute renal failure, cerebrovascular accident) and minor (e.g., urinary tract infection, paralytic ileus, electrolyte deficiencies) [13, 17, 32].

### 2.4. Glucose Control during Perioperative Period in Neurosurgery

Clinical consensus regarding perioperative glucose control in critically ill patients is lacking, especially in the neurosurgical population [12–14, 16, 30, 33]. There are no clinical studies in neurosurgical patients that have demonstrated significantly improved outcome with IIT, and those data available were collected from retrospective or small studies [32, 34, 35]. Furthermore, the heterogeneity of pathologies in neurologic surgery suggests caution in the generalization of other study results to this patient population: peripheral and cerebral glucose values do not correlate or correlate inversely; a normal cerebral glucose level is poorly defined, and the optimal level in the presence of anesthesia or brain pathology is unknown [12–14, 16, 30, 33]. In addition, no data exist to guide any type of differential management with IIT or fluid therapy in neurosurgical patients with or without preexisting DM. 

### 2.5. Preoperative Management: Patient Evaluation

#### 2.5.1. Patient Evaluation

Careful preoperative evaluation is essential in patients with DM to identify previously unknown complications and to manage comorbidities [14, 15, 30]. For elective surgery, it is prudent to organize a multidisciplinary team. There are neither guidelines nor large-scale trials that support one treatment plan. Blood glucose control in the perioperative period must be approached individually [14, 15, 30].

Before a planned surgical procedure, the patient's blood glucose should be as close as possible to that advocated by the American Diabetes Association [8, 14, 15, 30]. These targets include glycosylated hemoglobin (Hgb A1C) <7.0%, average preprandial plasma glucose between 90 and 130 mg/dL (5.0 and 7.2 mmol/L), and average postprandial plasma glucose <180 mg/dL (10.0 mmol/L) [8, 14]. Elective surgical procedures should be scheduled early in the day for patients with DM [15]. It may be advisable to delay elective surgery until satisfactory glucose control has been obtained. Glycemic control can typically be achieved with an insulin drip within a few hours. Cardiovascular disease can manifest atypically, occur at a relatively young age, and remain asymptomatic in patients with DM. The neurosurgeon and neurointensivist should seriously evaluate symptoms such as chest pain, exertional dyspnea, and orthopnea. Electrocardiography and stress testing with or without cardiac imaging may be warranted in high-risk patients with multiple risk factors. Patients with peripheral or cardiac autonomic neuropathy are prone to intraoperative hypotension, perioperative cardiac arrhythmia, gastroparesis, hypoG unawareness, and loss of glucose counterregulation [15]. The presence of resting tachycardia, orthostatic hypotension and loss of heart rate variability signal the potential for intraoperative problems. Serum creatinine level may not be a sensitive indicator of true kidney function in elderly patients with DM. A 24-hour urine collection may be indicated when there is an elevated serum creatinine level, proteinuria, or concomitant long-standing or poorly controlled hypertension. Insulin action is prolonged in renal impairment, promoting unpredictability of blood glucose and hypoglycemia. A practical way of action is reported in Table 2. It should be remembered that type II diabetics are vulnerable to an exaggerated variability in blood glucose levels possibly because surgical stress augments insulin resistance [14, 18, 19].

#### 2.5.2. Hyperglycemia Management: Pharmacologic Agents

**Antidiabetic Agents (ADA)**

The characteristics of currently available ADA, including the mechanism of action, duration of effects, dosing, and adverse effects, have been described in detail elsewhere [36]. Metformin and sulfonylureas should be withheld 24 hours before surgery [14, 36]. The long-acting sulfonylureas (e.g., chlorpropamide and glyburide) can cause prolonged hypoG and should be withheld for 48 to 72 hours [14, 36, 37]. Lactic acidosis is a rare but serious side effect of metformin, especially in elderly persons with compromised kidney function [36, 37]. Metformin may be restarted 48 hours after surgery, with the first meal, provided that hemodynamic stability is maintained and kidney function remains normal [14, 15, 30]. The thiazolidinediones (TZDs (e.g., rosiglitazone, pioglitazone)) are insulin-sensitizing agents that can cause fluid retention, intravascular volume expansion, and dilutional anemia [36, 37]. They can trigger pulmonary edema and congestive heart failure in susceptible patients, especially when used in conjunction with insulin. For this reason, they are contraindicated in patients classified as New York Heart Association class 3 and 4 congestive heart failure [36, 38]. TZDs should be restarted only after postoperative recovery is complete and there is no evidence of cardiopulmonary compromise or fluid overload [14, 15, 30, 36, 37]. The recently introduced incretin-based treatments are becoming popular as add-on medications in patients who do not achieve glucose goals with traditional oral therapy [36, 37]. These include exenatide, administered by daily subcutaneous (SC) injection, and the oral agent sitagliptin [36, 37]. Patients should discontinue these drugs 24 to 36 hours prior to surgery and restart the oral antidiabetics after discharge from the hospital [14, 36, 37].

In conclusion, ADAs do not have any use in critical pathologies, particularly during the perioperative period because of their unpredictable pharmacokinetics and pharmacodynamics [36, 37]. They interact with many drugs and their metabolism is influenced by increased capillary permeability or by hepatic and/or renal dysfunction [14, 36, 37]. Furthermore, these drugs act by promoting the secretion of insulin and/or by increasing the functionality of its receptors. During surgery both mechanisms are antagonized secondary to the stress response that decreases insulin secretion and increases resistance to insulin receptors [17, 39–41]. Finally, ADA can induce a more severe, prolonged, and often lethal hypoG in neurocritical patients [6, 7, 36, 37, 42].

**Insulin**

Insulin remains the mainstay of treatment for inpatients with perioperative hyperG. It is powerful, immediately effective, and has few contraindications or drug interactions. Insulin is the preferred medication in critically ill patients and in those with hepatorenal disease, cardiovascular limitations, or hemodynamic compromise [8, 14, 15].

### 2.6. Intraoperative Glucose Management and Postoperative Care

hyperG during neurosurgical procedures is best managed with a continuous IV insulin infusion. For an insulin drip, 100 U regular insulin can be added to 100 mL of IV fluid, thus achieving a concentration of 1 U/mL. Insulin is then given via infusion at a calculated rate. Alternatively, an empirical starting rate is 0.02 U/kg/h, titrated subsequently to achieve the goal glucose range. The infusion should be started well in advance of the procedure (preferably 2 to 3 hours) to allow titration to the desired glucose range. Hourly glucose readings are done intraoperatively; the insulin rate is adjusted to maintain the blood glucose within the target range. Insulin drip corrections are based on diverse monitoring schemes [8, 14, 15, 17]. The reactive approach delays insulin therapy until hyperG appears, while the proactive approach maneuvers blood glucose into a predefined target range [43]. Tables 3 and 4 report the reactive and proactive algorithms, respectively [44]. Another approach is to take initial measured blood glucose values and multiply them with a predefined multiplier. This approach has been shown to shift plasma glucose into a predefined target range within a few hours. Such a protocol can be applied manually as a standard column-based protocol (paper protocol) or can be driven by computer-guided support [45]. A recent study has shown that the computer-based algorithm resulted in tighter glycemic control without an increased risk of hypoglycemic events compared to the standard paper protocol [46].

Patients who are undergoing elective surgery and whose blood glucose is well controlled (as reflected by fingerstick readings and A1C values) without the use of insulin in the outpatient setting may not require an insulin drip and can be managed with SC supplemental insulin therapy [14, 15].

Postoperatively, the insulin infusion is continued at the physician's discretion. In the presence of stable blood glucose, insulin drip is converted to a subcutaneous insulin regimen with a basal insulin dose and bolus/nutritional insulin dose. Basal insulin can be given as glargine, levemir, or neutral protamine Hagedorn (NPH). Bolus/nutritional insulin can be either given as regular insulin or as one of the insulin analogs such as glulisine, aspart, or lispro. Regular insulin is the preferred drug if the patient continues to be on enteral tube feeding. However, if the patient resumes his regular diet, insulin analogs are advantageous due to their immediate effect [14, 15].

When transitioning from IV to SC insulin, the drip should continue and overlap with the first SC dose of long-acting (basal) insulin for two to four hours. Failure to overlap IV and SC insulin can result in extreme hyperglycemia or diabetic ketoacidosis (DKA). Usually, ADA are restarted after the first proper food intake if there are no contraindications [14, 15, 36, 37].

In the presence of reactive hypoG, we recommend the scheme depicted in Table 5.

#### 2.6.1. Impact of Anesthetics Agents on Systemic and Cerebral Metabolism of Glucose

Anesthetics influence systemic and brain metabolism. Although most of these agents have depressor effects on brain oxygen and glucose consumption, their actions differ from one another [47–51].

**Volatile Anesthesia**

Isoflurane diminishes cerebral metabolism, preserving high-energy phosphates and inducing extracellular glucose elevation [47, 48, 51]. Conflicting data have been reported about isoflurane's effect on lactate production ranging from nonaccumulation to elevation of 300% [51]. However, a microdialysis study reported that lactate elevation is associated with a concomitant pyruvate elevation without changes in the lactate/pyruvate (L/P) ratio and in glucose or glutamate concentrations [51]. Furthermore, isoflurane decreases insulin secretion predisposing the patient to hyperG [50, 52].

**Intravenous Anesthetics**

*Barbiturates (BBT)* are depressors of global metabolism without production of lactate accumulation. They have no relevant effects on systemic glucose regulation [51].

*Propofol *causes a minimal elevation in extracellular glucose [51]. Unlike isoflurane, it stimulates insulin secretion and, therefore, is less likely to generate hyperG [52].

*Ketamine* produces a mild-to-moderate rise in cerebral oxygen and glucose consumption. It also increases cerebral lactate levels moderately [51].

*Opioids* do not have major effects on glucose metabolism.

*Etomidate* inhibits ACTH secretion and could, therefore, induce hypoG [47–51].

*Benzodiazepines* decrease cerebral metabolic consumption globally, maintaining dose-dependent coupling with the CBF [47–51].

*Neuroaxial anesthetics* block the autonomic and neuroendocrine response to hypoG when used in spinal or epidural anesthesia [50].

### 2.7. Target Blood Glucose Levels in Neurosurgery: Clinical Studies

#### 2.7.1. Intensive Insulin Therapy

In 2001, Van Den Berghe published the results of a new therapeutic modality directed to keep blood glucose between 80 and 110 mg/dL (4.4 and 6.2 mmol/L) called “intensive insulin therapy” (IIT) [5]. This was a prospective, randomized, single-center trial including 1564 patients of whom 60% were postcardiac surgery. Patients randomized to IIT were compared with a group that had blood glucose targets between 180 and 200 mg/dL (10.0 and 11.1 mmol/L). A significant decrease in mortality (8% versus 4.6%, *P* < 0.04) was observed mainly in septic patients with multiorgan dysfunction and more than five days in the critical care unit. Additional benefits were decreased incidence of infections and reduced rates of mechanic ventilation, hospital mortality, polyneuropathy, blood transfusions, and dialysis in patients with acute renal failure [5].

In 2009 a similar trial was published in different critical patient populations with controversial results. The recently reported findings from the multinational Normoglycemia in Intensive Care Evaluation—Survival Using Glucose Algorithm Regulation (NICE-SUGAR) trial are particularly relevant [53]. In this prospective, randomized, multicenter trial, intensive and conventional blood glucose control were compared in 6,104 patients in the intensive care unit with different medical and surgical pathologies. IV insulin was used to achieve a blood glucose level of 81 to 108 mg/dL (4.5 to 6.0 mmol/L) in the intensive group and 144 to 180 mg/dL (8.0 to 10.0 mmol/L) in the conventional group. At 90 days after admission in the ICU, mortality in the intensive group was significantly higher (27.5% versus 24.9%; *P* = 0.02), and was as severe as hypoG incidence (6.8% versus 0.5%, *P* < 0.001) [53].

A consensus statement of the American Association of Clinical Endocrinologists and the American Diabetes Association [8] has recommended revising glucose targets. In critically ill patients, start treatment at a threshold of >180 mg/dL (>10.0 mmol/L), preferably with IV insulin therapy, and maintain the glucose level between 140 and 180 mg/dL (7.8 and 10.0 mmol/L). Greater benefit may be obtained at the lower end of this range. Glucose concentrations <110 mg/dL (6.0 mmol/L) are not recommended [8]. However, these goals should be flexible and individualized to the particular patient and the clinical circumstances [8]. Persistently elevated readings indicate that the treatment regimen must be adjusted or changed and should alert the treating physician of the need to explore the possible reasons for hyperG [8].

#### 2.7.2. Mixed Neurosurgical Populations

Two retrospective studies, with methodological limitations from Brazil and Australia, came to the same conclusion in neurosurgical patients [54, 55]. IIT was associated with a greater risk of developing hypoG without reducing mortality or improving neurologic functional status [54, 55]. In a prospective, randomized controlled trial, Bilotta et al. [32] analyzed the safety profile and efficacy of an aggressive blood glucose control [80–110 mg/dL (4.4–6.2 mmol/L)] versus control management [180–216 mg/dL (10.0–12.0 mmol/L)] in an unselected neurosurgery population. Early enteral or parenteral feeding was started with standard regimes. The therapeutic protocol was followed until patients were discharged from the ICU or until the second week after surgery. Although the length of stay in the ICU and infection rate were lower in the group of patients receiving intensive treatment, the number of hypoG episodes were higher, without any difference in mortality or functional results at six months after surgery [32].

#### 2.7.3. Aneurysmal Subarachnoid Hemorrhage and Intracerebral Hemorrhage

Hematoma evacuation and aneurysm clipping are common reasons for presentation to the operating room. Many patients who present for clipping have already experienced some degree of aneurysmal subarachnoid hemorrhage or intracerebral hemorrhage (SAH/ICH). After SAH/ICH regional abnormalities in CBF, posthemorrhage edema, vasospasm, and increased ICP all predispose the brain tissue to ischemia [56–61]. Despite the apparent link between hyperG and symptomatic vasospasm, infarct size, and outcome, the few studies of IIT in these populations have failed to demonstrate a significant difference in outcome with tight glucose control [61–63]. All but one of the studies were retrospective, and target ranges were generally under 140 mg/dL (7.8 mmol/L). The groups treated with IIT developed significantly fewer infections than the control group (27% versus 42%;  *P* < 0.001), but the benefit was a reduction in the prevalence of postsurgery vasospasm. Neither mortality nor functional outcomes were affected [34]. Recently Latorre et al. [64] compared glucose management in two different time periods in patients with aneurysmal SAH in a retrospective analysis. Before 2003, blood glucose was corrected if the levels were >200 mg/dL (>11.1 mmol/L); after 2003, blood glucose was controlled more strictly and aggressively maintaining levels between 80 and 140 mg/dL (4.4–7.8 mmol/L). There were no differences in final outcomes between the groups, but there was a tendency for improvement in patients treated to a plasma glucose range of 80–140 mg/dL (4.4–7.8 mmol/L) [64]. In general, hyperG is linked with worse outcome. While insulin therapy in SAH patients was shown to effectively control plasma glucose levels, plasma glucose control is not necessarily reflective of cerebral glucose such that very tight glucose control may lead to neuroglycopenia. However, tight glycemic control is associated with an increased risk for hypoG which was linked to worse outcome [57].

These data suggest that a benefit from tighter glucose control during acute episodes of ischemia (such as with temporary clip application or aneurismal rupture) would be mechanistically plausible. It seems appropriate to consider stricter control when acute, focal ischemia is occurring or anticipated, but continuation of tight control into the postoperative phase is not supported by the literature.

In ICH, admission hyperglycemia is associated with increased 30-day mortality and worse functional outcome [65–67]. Decline in serum glucose concentration correlated with reduction in proportion of subjects with hematoma expansion and decrease of poor clinical outcome [65]. However, the targets for glycemic control are unclear, and there is increasing evidence that “tight” glycemic control with insulin infusion can be associated with a critically low cerebral extracellular glucose concentration after brain injury [68]. Until further data become available, systemic glucose levels should not be treated in the acute phase after ICH unless >180 mg/dL (>10 mmol/L) [10], although it seems reasonable to treat patients with insulin when their peripheral glucose value is greater than 150 mg/dL (>8.3 mmol/L) [16, 44].

#### 2.7.4. Severe Traumatic Brain Injury

A substantial body of literature exists regarding glucose management in patients with TBI [35, 69–71]. It is clear that TBI represents a continuum of injury with heterogeneous changes in regional brain function and glucose metabolism. Under certain circumstances, cerebral hypoG may be a significant concern that complicates management. Thus, these studies do not show a relevant clinical benefit in using IIT in severely head-injured patients [35, 69–71].

#### 2.7.5. Spine Surgery

There are no specific studies of perioperative glucose management in patients undergoing spinal surgery for tumor or correction of scoliosis. In 1989, Drummond and Moore [72] reported that, in rabbits, glucose administration prior to temporary spinal cord ischemia dramatically increased the likelihood of paraplegia. Woodworth et al. [73] reported, in a retrospective study, that a single preoperative episode of hyperG in patients undergoing intramedullary spinal tumor resection correlated with a likelihood of poor postoperative ambulatory function [73]. If the mechanism of anticipated spine injury is assumed to be focal ischemia, it seems reasonable to extend the models of acute focal ischemia to the spine and conclude that pronounced hyperG immediately prior to hardware manipulation has the potential to worsen the extent of injury. Judicious use of insulin to maintain blood glucose less than 150 mg/dL (8.3 mmol/L) before and during periods of potential ischemia is prudent and safe.

#### 2.7.6. Neurosurgery for Tumors and Intracranial Masses

There are no specific studies of perioperative management of glucose in patients with intracranial mass. Most patients presenting for tumor resection receive perioperative corticosteroids. This therapy is associated with increased plasma glucose and also with decreased cerebral glucose utilization [30]. In a retrospective study, McGirt et al. [74] showed an association between persistent postoperative hyperG and mortality in patients undergoing tumor resection. The use of glucose-containing solutions to replace water deficit in the perioperative period should be avoided in patients undergoing resection of a pituitary mass due to the high risk of developing central diabetes insipidus during the late intraoperative, or, more commonly, postoperative period [75, 76].

#### 2.7.7. Interventional Neuroradiology (Tissue Plasminogen Activator and Vasospasm)

With increasing frequency, patients present to the interventional radiology suite for aneurysm coiling, injection of intra-arterial recombinant tissue plasminogen activator (rTPA) after acute stroke, and intra-arterial vasodilatory agents to treat symptomatic vasospasm. These are clinical circumstances in which a very acute ischemic episode has occurred, and the clinical intervention may be accompanied by sudden reperfusion. Non-DM patients with acute middle cerebral artery (MCA) ischemia who received IV rTPA had larger cerebral stroke volume, and worse 28-day outcome if hyperG (>180 mg/dL) was present [77]. However, glucose was measured on admission and immediately treated with insulin if elevated. One intriguing finding was that two patients in the hyperglycemic group, who were treated with insulin prior to emergency department arrival, had outcomes similar to the normoglycemia group [77]. In a cohort of 1083 stroke patients, Poppe et al. [78] reported that admission hyperG [>144 mg/dL (>8.0 mmol/L)] was associated with greater risk of ICH, mortality, and poor 90-day outcome after intravenous tissue plasminogen activator (TPA). Similar results are confirmed in other studies [79, 80]. Most of these data are retrospective and based on a single glucose measurement in a largely diabetic population, so clear conclusions cannot be drawn. However, it seems prudent to obtain peripheral blood glucose measurements in every patient who presents for intra-arterial thrombolysis or treatment of symptomatic vasospasm and to treat values greater than 144 mg/dL (>8.0 mmol/L) with insulin during the immediate periprocedure period.

## 3. IIT: Systematic Review and Published Guidelines

Recently a systematic review was published from a meta-analysis of 21 trials in heterogenic populations of critically ill patients, including stroke and head trauma [81]. On the basis of this systematic review, the American College of Physicians recommended not using IIT under any circumstances in hospitalized patients [82]. Actually, tight blood glucose control does not have any solid evidence for its implementation in the perioperative neurosurgical period or in victims of any cerebral injury from any cause [12, 13, 33].

### 3.1. Intracerebral Hemorrhage

Current guidelines from the American Heart Association recommend insulin treatment for patients with blood glucose levels >185 mg/dL (>10.3 mmol/L) and possibly even those with levels >140 mg/dL (>7.8 mmol/L: evidence Class IIa, Level of Evidence C) [83]. In contrast, ICH guidelines endorsed by the major European stroke and neurological societies suggest maintaining blood glucose below 300 mg/dL (16.7 mmol/L) [39].

### 3.2. Subarachnoid Hemorrhage

Recently published guidelines from the American Heart Association emphasize the importance of avoiding hyperG in patients with aneurysmal SAH, but without providing specific recommendations on target glucose levels [40].

### 3.3. Traumatic Brain Injury

Guidelines from the Brain Trauma Foundation [41] and the European Brain Injury Consortium [84] highlight the association of hyperG with worse prognosis after severe brain trauma, but these documents do not specify which glucose level should be considered as a trigger for initiating insulin therapy.

### 3.4. Acute Spinal Cord Injury

Recently, the consortium for Spinal Cord Medicine published practical guidelines for the acute management of spinal cord injury in adults online (http://www.pva.org). They recommend maintaining serum glucose values between 80 and 110 mg/dL (4.4 to 6.2 mmol/L), albeit acknowledging a low level of evidence to support such recommendation.

## 4. Glucose Variability and Monitoring

There are no guidelines or recommendations establishing the method of choice and optimal monitoring frequency. The American Diabetes Association [8] suggests “frequent” monitoring, while the “Surviving Sepsis Campaign” suggests monitoring every 1-2 hours [85].

Glucose levels fluctuate continuously, and minimal variations are induced by multiple factors, such as stress, pain, trauma, surgery, and drugs [86]. Wide variations constitute an independent mortality predictor in critical patients, perhaps reflecting the severity of the insult [87]. To date the reason for this phenomenon has not yet been elucidated. One hypothesis is that increased glucose variability is caused by oxidative stress secondary to brain damage [88].

Few studies have elucidated the time course of blood glucose after brain injury. In ischemic stroke, Baird et al. [89] observed hyperG at admission in 35% of his patients; 43% of the patients continued to have elevated levels for 72 hours. Of the patients with normal levels at admission, 46% develop hyperG later. In a similar population, Allport et al. [90] identified an early (within 8 hours after stroke onset) and later (48–88 hours) hyperG, and Godoy et al. [44] identified four different evolutional patterns in patients with spontaneous ICH, showing a different impact on early mortality.

### 4.1. Monitoring

There are different forms of monitoring, but all use enzymatic reactions (glucose oxidase or dehydrogenase). It is easy to obtain arterial or venous blood samples; capillary or subcutaneous samples must be obtained using sensors inserted in the abdominal wall [89, 90]. Plasma glucose samples are the gold standard (about 11% higher than obtained by whole blood) by conventional laboratory methods [8], but obtaining them requires extreme precautions to avoid contamination with parenteral solutions. Some blood gas analyzers that allow obtaining fast and reliable glucose values are not always available in the ICU or operating room. The accuracy of a blood glucose monitor can be affected by several factors: type of blood glucose strip and monitor, very low glucose values, presence of edema, anemia, and peripheral hypoperfusion [8, 15, 17]. Devices used for blood sugar measurements are calibrated to obtain plasma glucose with normal hematocrit values [17]. If hematocrit decreases, the blood glucose level can be overestimated by up to 30% [15]. Interfering substances, such as drugs commonly used in critically ill patients, may account for additional causes of error in determining blood glucose concentrations (Table 6). Subcutaneous sensors determine blood glucose levels on a continuous basis (every few minutes) by measuring glucose levels of interstitial fluid. These continuous systems must be calibrated with a traditional blood glucose measurement (using current technology). Glucose levels in interstitial fluid lag behind blood glucose values [15, 17]. Furthermore, they have not been validated for routine utilization in critically ill patients or intraoperatively [89, 90]. Vascular sensors that allow continuous monitoring are in development.

### 4.2. Nutrition for Blood Glucose Control

A strategy of blood glucose control should include a nutrition protocol with the preferential use of the enteral route [8, 14, 15, 35, 38, 91]. Initiating blood glucose control without adequate provision of calories and carbohydrates will increase the risk of hypoG [8, 14, 15, 35, 38, 91]. This strategy of strict blood glucose control should be carefully coordinated with the level of nutritional support and metabolic status, which changes frequently in neurocritically ill patients. A recent study, evaluated the nutrition protocol's influence on brain metabolism using microdialysis in patients with SAH [38]. Two hours after 250 Kcal by nasojejunal tube feeding, there were simultaneous increments in glucose levels in the blood and cerebral extracellular space without a change of glutamate concentration or the L/P ratio [38]. Stress hyperG exacerbates the disorders in gastric motility as a result of several factors such as cytokines produced by inflammation, oxidative stress, vasoactive intestinal peptides, splanchnic hypoperfusion, and drugs such as phenytoin, steroids, and opioids [91]. Acute gastroparesis differs from diabetic gastroparesis in that it is reversible and sensitive to prokinetics [91]. Acute gastroparesis causes an interruption of appropriate feeding, which contributes in a wider variability in blood glucose levels, showing an increase in insult severity. However, solid clinical evidences and practical considerations are not provided for nutrition support regimens to minimize stress hyperG and assist glucose management. Actual guidelines are based on small patient series and expert opinion only [91]. In Table 7 we show current recommendations (experts opinion) for good glucose control during nutritional support [91].

## 5. Management of Hypoglycemia

No specific trial has addressed the question of what the best management of hypoG is, while experimental data suggest new strategies to reduce brain damage due to severe hypoG [92]. Nowadays, IIT is the most prevalent cause of the development of hypoG. Available evidence suggests that hypoG must be avoided by all means and must be treated urgently, because it is closely linked to worse short- and long-term outcomes [6–8, 42]. We recommend implementing alerts when blood glucose values are near 90 mg/dL (5.0 mmol/L) in neurocritically ill patients because microdialysis monitoring showed that even normal blood glucose levels can also induce cellular derangement in brain-injured patients [59, 68].

## 6. Conclusions and Future Directions: What Is the Optimal Glucose Level?

The optimal range of blood glucose levels in neurosurgical and neurocritically ill patients has not been determined and remains controversial. No consensus exists on blood glucose level goals for the perioperative period; however, several organizations have established general targets for neurocritically ill and neurosurgery patients [12, 16]. The question of optimal blood glucose goals cannot be answered with certainty, especially in neurocritically ill patients [12, 13, 16, 30, 33]. The brain is very vulnerable to extreme blood glucose level variations. It was demonstrated that an energy crisis may even occur with blood glucose levels within normal range [59, 68]. Therefore, it would be essential to know what the safe lower limit is. However, neither PET scanners nor microdialysis are available in every ICU. The American Diabetes Association and the American Association of Clinical Endocrinologists [8], based on the available evidence, set an upper limit at 180 mg/dL (10 mmol/L), at which insulin therapy should be started. This would also propose to maintain blood glucose levels between 140 and 180 mg/dL (7.8–10.0 mmol/L) [8] in critically ill patients and in the perioperative period. The available clinical data do not support tight glucose control with IIT in this critically ill subpopulation [12, 16]. Two meta-analyses of all patient types treated with IIT drew similar conclusions [81, 82].

**Figure 2:**
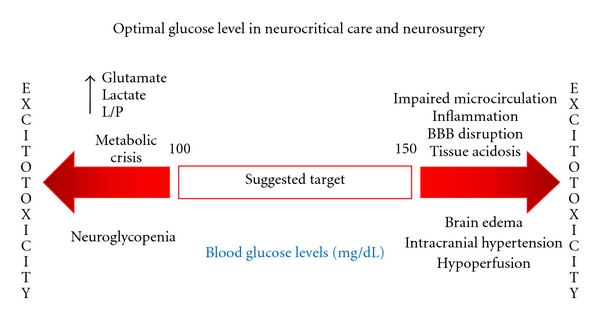
Suggested glycemic targets during acute brain injury/neurosurgery. Where L/P: Lactate/Pyruvate and BBB: Blood–brain barrier.

**Figure 3:**
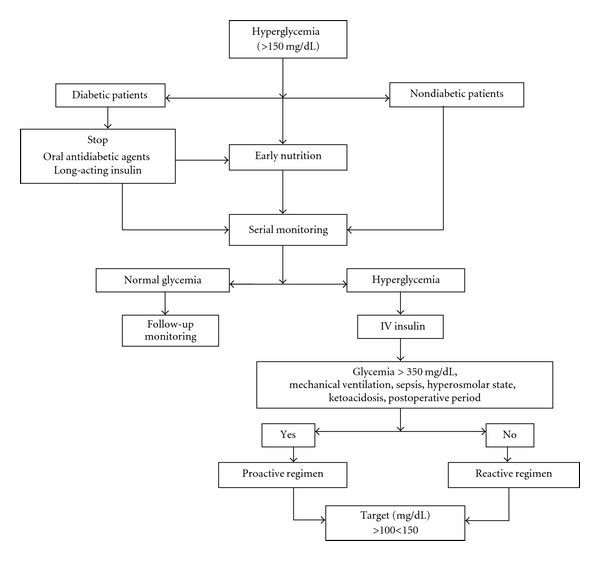
Algorithm proposed for hyperglycemic management.

Figure 2 outlines where blood sugar levels should be kept in patients with acute brain injury, while Figure 3 shows our proposal for an algorithm in hyperglycemia management to use in neurocritically ill patients.

Ongoing and future research promises to clarify the present muddled picture. Examples include stratification of neurologic-injury-based protein and biochemical biomarkers and identifying potential high-throughput strategies that will allow one to individualize disease management.

Further studies on the multimodal effects of insulin via modulation of signaling pathways, such as inflammation, cell adhesion, and activity of glucose transporters and pyruvate metabolism enzymes, will have to be conducted. Investigation of agents other than insulin, such as glucagon-like peptide-1 (GLP-1), for glucose-lowering effects may demonstrate a reduced rate of hypoG or other beneficial metabolic effects. Microdialysis studies in patients undergoing IIT therapy could provide important insight into regional alterations of glucose metabolism in injured brain tissue. Neurocritically ill patient populations are heterogeneous, and data interpretation and generalization has to be done with caution. We need a better understanding of all these pathophysiological processes before adoption of IIT.

Finally, special attention should be drawn to the optimal technology for accurate, reliable, and rapid glucose measurement. Closed-loop continuous glucose control systems should be developed. New technologies may facilitate the avoidance of hypoG under an IIT regimen and the development of IIT protocols that can be individualized to the specific metabolic state of the patient under treatment.

### 6.1. Key Points

- hyperG is frequent in acute neurological diseases in the perioperative period both in diabetics and nondiabetics. hyperG [>150 mg/dL (>8.3 mmol/L)] is associated with poor outcome, but causality has not been definitively demonstrated.
- Extreme hypoG and hyperG episodes must be avoided.
- It is recommended to maintain blood glucose levels between 140 and 180 mg/dL (7.8–10.0 mmol/L).
- Oral antidiabetic agents have no place in acute and critical situations.
- Regular intravenous insulin is preferred to lower blood glucose levels.
- IIT has no benefits.
- Frequently and routinely monitor glucose levels.
- Implementation of an institutional multidisciplinary management protocol is recommended.
- Therapy must be accompanied by adequate nutritional support.

## Figures and Tables

**Table 1:** Hypoglycemia causes in neurocritical care patients.

(i) Starvation
Prolonged hospitalization
Pregnancy
(ii) Drug Induced
Insulin (intensive insulin therapy)
Hypoglycemic agents
Alcohol
Etomidate
Beta blockers
Cyprofloxacin
Salicylates
Enalapril
Warfarin
Acetaminophen
(iii) Sepsis
(iv) Renal dysfunction
(v) Hepatic dysfunction
(vi) Endocrine
Hypopituitarism
Adrenal insufficiency
Hypothyroidism
Hyperinsulinemia: parenteral nutrition
(vii) Idiopathic
(viii) Iatrogenic

**Table 2:** Preoperative evaluation of the neurosurgical patient.

*(i) Type II diabetics, noninsulin dependents:* stop all oral
antidiabetic agents. In elective surgeries, hold oral antidiabetic
agents 24 hours before intervention especially those such as
chlorpropamide with a long half-life.
*(ii) Type II diabetics, insulin dependents:* for elective short
procedures (<2 hours), suspend regular insulin. Administer
only 2/3 of the long-acting insulin (NPH) or give the full dose of
basal insulin (glargine, levemir), and start nutrition 12 hours after
surgery.
*(iii) Type I or II diabetics:* for long surgeries or emergencies:
only use regular insulin according to unit's protocol.

**Table 3:** Reactive regimen (according to monitorized values). The measurement unit used for indicating the concentration of blood or plasma glucose can either have a weight dimension (mg/dL) or a molarity (mmol/L).

Glucose value		Insulin dose
mmol/L*	mg/dL^†^	IU
≤8.3	≤150	—
8.4–11.1	151–200	5
11.2–13.9	201–250	10
13.93–16.7	251–300	15
16.71–19.4	301–350	20

Exact conversion of glucose values from mg/dL to mmol/L and vice versa are as follows:

*mmol/l = mg/dL × 0.0555,

^†^mg/dL = mmol/L × 18.0182

**Table 4:** Proactive regimen: dilute 100 U of insulin in 100 mL of isotonic saline solution 0.9% (1 U = 1 mL). Administer via infusion pump according to the following scheme. The measurement unit used for indicating the concentration of blood or plasma glucose can either have a weight dimension (mg/dL) or a molarity (mmol/L).

Glucose value		Insulin infusion rate
mmol/L	mg/dL	IU/h
8.3–9.4	150–169	2
9.43–11.0	170–199	3
11.1–13.8	200–249	4
13.9–16.6	250–299	6
16.7–22.1	300–399	8
22.2+	400+	10

Exact conversion of glucose values from mg/dL to mmol/L and vice versa are as follows:

*mmol/L = mg/dL × 0.0555,

^†^mg/dL = mmol/L × 18.0182.

**Table 5:** Hypoglycemia management.

Administer hypertonic dextrose (50%) according to the following
formula:
(100 − glycemia) × 0.3 = mL in IV bolus
Check plasma glucose every 30 minutes
If glucose < 60 mg/dL (3.3 mmol/L), repeat the IV bolus step

Overcorrection will be avoided in all cases.

**Table 6:** Capillary glucose monitoring with test strips: factors interfering with correct determination.

(i) Factors overestimating the accurate value (false rise)
Anemia
Paracetamol
Dopamine
Mannitol
Hyperuricemia
Vitamin C
Jaundice
Immunoglobulins
(ii) Factors underestimating the accurate value (false drop)
High hematocrit (polycythemia—COPD)
Hypoperfusion
Noradrenaline (high doses)
Edema
Hypoglycemia
paO_2_ > 100 mmHg

**Table 7:** Recommendations for adequate nutritional support during glucose control protocol.

(i) Avoid excessive caloric intake especially carbohydrates
(ii) No more than 25–30 calories per kg body weight per day
(iii) 25% of intake in the form of lipids
(iv) Insulin therapy according to needs
